# Distribution pattern of the snail intermediate host of schistosomiasis japonica in the Poyang Lake region of China

**DOI:** 10.1186/s40249-019-0534-8

**Published:** 2019-03-29

**Authors:** Fei Hu, Jun Ge, Shang-Biao Lv, Yi-Feng Li, Zhao-Jun Li, Min Yuan, Zhe Chen, Yue-Ming Liu, Yue-Sheng Li, Allen G. Ross, Dan-Dan Lin

**Affiliations:** 1 Jiangxi Provincial Institute of Parasitic Diseases, No. 239, First Gaoxin Rd., Gaoxin District, 330096 Nanchang, Jiangxi Province People’s Republic of China; 2 Molecular Parasitology Laboratory, Infectious Diseases Division, QIMR Berghofer Medical Research Institute, Brisbane, Australia; 3 Menzies Health Institute Queensland, Griffith University, Gold Coast, QLD Australia

**Keywords:** Schistosomiasis, China, Poyang Lake, *Oncomelania hupensis*, Snail control, GIS mapping, Hotspot, Disease elimination

## Abstract

**Background:**

With the closure of the Three Gorges Dam in 2003 the hydrology of Poyang Lake was altered dramatically leading to significant changes in the environment. In order to assess the impact on schistosomiasis this study assessed the spatial and temporal patterns of the snail intermediate host, *Oncomelania hupensis* in the Poyang Lake tributaries. The results of the study have important implications for future snail control strategies leading to disease elimination.

**Methods:**

The marshland area surrounding Poyang Lake was divided randomly into 200 × 200 m vector grids using ArcGIS software, and the surveyed grids were randomly selected by the software. The snail survey was conducted in each selected grid using a survey frame of 50 × 50 m with one sideline of each grid serving as the starting line. No less than ten frames were used in each surveyed grid with Global Positioning System (GPS) recordings for each. All snails in each frame were collected to determine infection status by microscopy. Altitude data for all frames were extracted from a lake bottom topographic map in order to analyze the average altitude. All snail survey data were collected and statistically analyzed with SPSS 20.0 software in order to determine the difference of the percentage of frames with living snails and mean density of living snails in different regions of Poyang Lake. The altitude of the snail-infested marshlands and snail dens were subsequently identified.

**Results:**

A total of 1159 potential snail sampling grids were surveyed, of which 15 231 frames (0.1 m^2^/frame) were investigated. 1241 frames had live *Oncomelania* snails corresponding to 8.15% of the total number of frames. The mean density of living snails was 0.463/0.1 m^2^ with a maximum of 57 snails per frame. The percent of frames with snails in the southern sector (8.13%) of Poyang Lake did not differ statistically from the north (8.21%). However, the mean density of live snails in the northern sector (0.164/0.1 m^2^) of the lake was statistically higher (*F* = 6.727; *P* = 0.010) than the south (0.141/0.1 m^2^). In the south of the lake, the elevation of snail-inhabited marshland ranged between 11 - 16 m, and could be further subdivided into two snail-concentrated belts at 12–13 m of elevation and 15–16 m of elevation respectively. In the north of the lake, the elevation of snail-inhabited marshland ranged between 9− 16 m with the elevation of 12–14 m being the snail-concentrated zone.

**Conclusions:**

The elevation of snail-infested marshlands in the Poyang Lake region ranged from 9 to 16 m. The snail distribution and habitat has moved north of the lake and to a lower altitude due to changes in the water level post dam closure. Based on the current geological features of the snail habitant focused mollusciciding should occur in snail dense northern regions with frequent bovine and human traffic. Targeting these identified ‘hotspots’ of transmission will assist in elimination efforts.

**Electronic supplementary material:**

The online version of this article (10.1186/s40249-019-0534-8) contains supplementary material, which is available to authorized users.

## Multilingual abstracts

Please see Additional file 1 for translations of the abstract into the five official working languages of the United Nations.

## Background

Scistosomiasis is considered a major public health in China and one of the diseases targeted for elimination [1]. *Oncomelania hupensis,* the sole intermediate host of *Schistosoma japonicum* in China, is vital for disease transmission. After more than 60 years remarkable achievements have been made in terms of schistosomiasis control in the People's Republic of China [2]. In 2015 China obtained the goal of schistosomiasis transmission control at the national level, and in five provinces/autonomous regions/municipalities (e.g., Shanghai, Zhejiang, Kuangtong, Fujian and Guangxi) the disease had been eliminated, while in Sichuan Province, schistosomiasis transmission has been interrupted [3].

Various measures of snail control play an important role in the global control of schistosomiasis and contribute to the great achievements that have been made in China to date. According to recent statistics the area of snail habitat nationally has decreased by 75% from 14.32 billion m^2^ in the 1950s to 3.57 billion m^2^ in 2016. The marshlands in the Poyang and Dongting lakes account for 95% of the present snail habitat [4]. The lake regions represent the last strong hold of the disease where the water levels cannot be adequately controlled and the snail intermediate host is widely distributed. The existence of vast snail habitats is a key factor that hampers the progress of schistosomiasis elimination in China and is one of the risks for periodic rebounds in disease transmission [5–8].

Jiangxi province is one of remaining highly endemic provinces in China. It has 0.787–0.810 billion m^2^ of snail-infested areas of which marshlands account for 97%. The vast majority of snails currently distributed in the Poyang Lake region are in newly developed snail environments post closure of the Three Gorges Dam [4, 9]. The human prevalence is less than 5 % in formerly highly endemic communities. Exposed populations live adjacent to the marshlands with occupational risks associated with fishing, farming, and washing clothes.

The vast marshlands of Poyang Lake are historically suitable for snail breeding. After closure of Three Gorges Dam in 2003 the hydrology in Poyang Lake has changed dramatically. The results of a water level surveillance survey conducted during the period of 1973–2011, at the Hukou Hydrological Station, showed that the mean water level of the lake was 13.1 ± 0.9 m before 2005 and decreased to 11.8 ± 0.9 m after 2006 [10]. At Xinzi Hydrological Station, the number of days when the water level was higher than 13 m has reduced, while increased when the water level was lower than 11 m. This indicates that lower water levels are coming earlier in the year and that the dry season is more prolonged [11, 12]. It has also become apparent that the distribution of snails and their habitats have changed due to lower water levels post closure of the dam [13]. Given the lack of information on the current temporal and spatial patterns of the intermediate host of schistosomiasis in the Poyang Lake region we carried out an environmental study in order to determine the distribution of *Oncomelania hupensis* in the marshlands surrounding Poyang Lake. With the development of the Global Positioning System (GPS), Geographic Information System (GIS) and spatial statistics, this has provided new and effective tools to explore the relation between *Oncomelania* snails, schistosomiasis, and the natural environment [14–17]. In this study, we specifically investigated the distribution of *Oncomelania* snails in the Poyang Lake region using GIS and GPS in order to ascertain the dynamic changes in snail distribution patterns at different elevations in the marshlands and to further define the snail habitat. The outcomes are important for national integrated schistosomiasis control strategies leading to disease elimination.

## Methods

### Study area

**Fig. 1:**
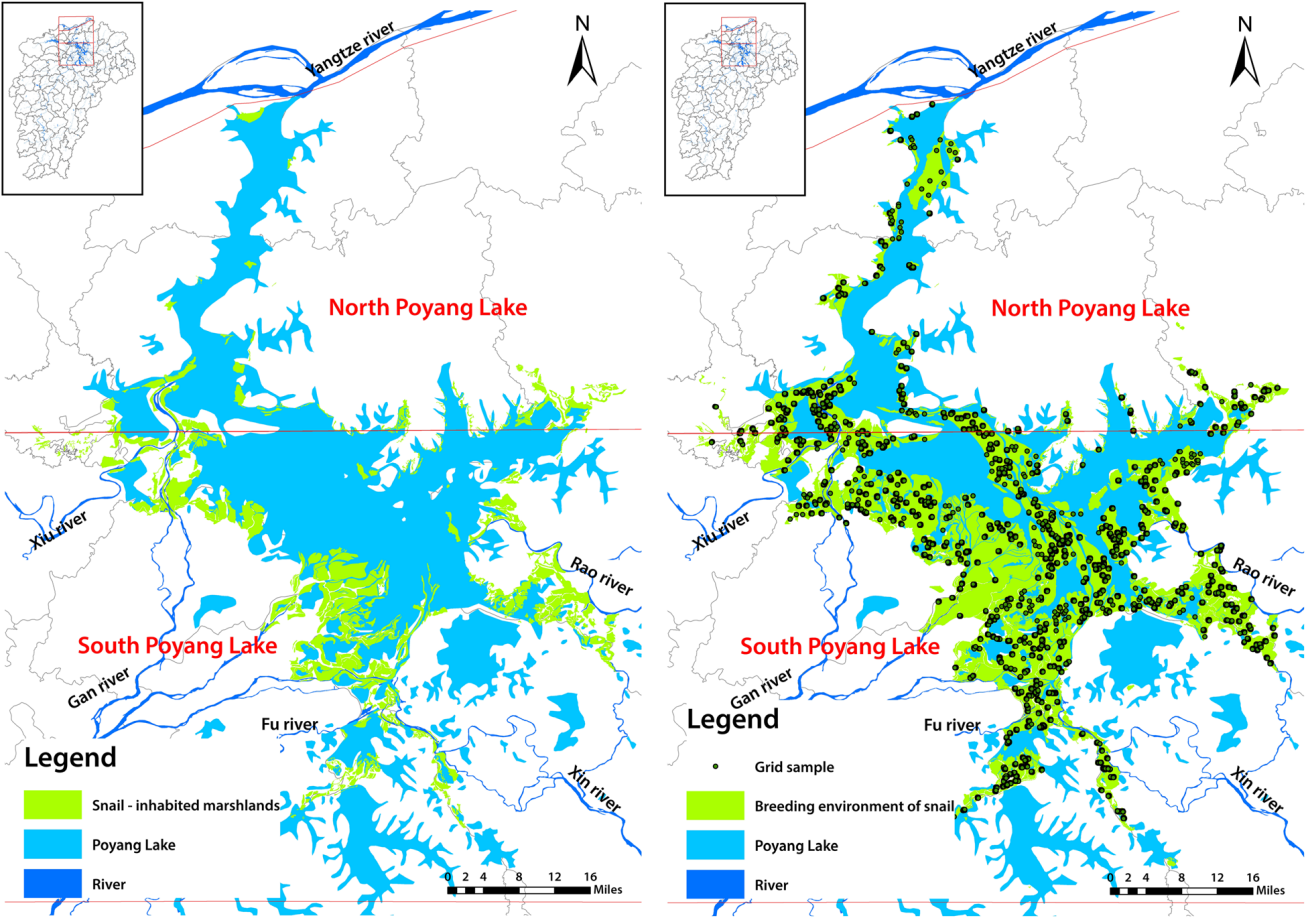
Left panel: Map of the study area in the Poyang Lake before the closure of the Three Gorges Dam closure in 2002. The green represent the snail-inhabited marshlands. Right panel: Geographical spatial map of the study area and spatial distribution of the sampling grid in Poyang Lake post the Three Gorges Dam closure in 2014. The dots represent grid samples for snail survey

The study was conducted in the Poyang Lake region of China which is situated in northern sector of Jiangxi Province [18]. It lies in a structural depression south of the Yangtze River with the coordinates of 28°11′ − 29°51′N, and 115°49′ − 116°46′E. It is fed by various rivers from Jiangxi, the most important being the Gan River, Wu River, Xin River, and Xiu River. Poyang Lake, the largest lake in China, drains into the Yangzte River at Hukou. The lake is a seasonal impounding and releasing lake, and the size of the lake between flood and dry season varies greatly every year from less than 1000 km^2^ in winter to more than 3000 km^2^ in summer [19, 20]. In summer as the water level rise the marshlands become submerged. In winter flood waters recede and the covered marshlands appear once again. As shown in Fig. 1, the lake is divided into two sections bounded by Songmen Mountain. The northern section is the waterway to the Yangzte River. The much larger southern section is main lake body [21]. There are three types of marshlands: sand land, mud land, and grass land, with 3130 km^2^ of total area and 10–16 m of elevation. There are few sand lands found at lower elevation and mainly located on both sides of river ways. The number of mud lands is greater and their elevations are below 12 m. The grass lands are primarily located in deltas where rivers enter the lake at elevations of 12–15 m [22] (Fig. 1).

### Sampling grids

The spatial map of the snail-infested marshlands was established using ArcGIS (10.2, ESRI, RedLands, USA) with WGS-84 geographic coordinate system and Transverse Mercator projection coordinate system [23]. 200 × 200 m vector grids were created on the spatial map of marshlands through Hawth’s Tools in ArcGIS software, and the surveyed grids were randomly selected by the software. The formula for calculating grid sample size was *n* = Z^2^P (1-P)/e^2^, with e = 0.03, *P* = 0.4713 and Z = 1.96 (data came from average density of living snails in surveillance locations of the province in 2014) [24]. Longitude and latitude center points for each sample grid were derived, and the database of sample grids was established according to the county-level administrative divisions.

**Fig. 2:**
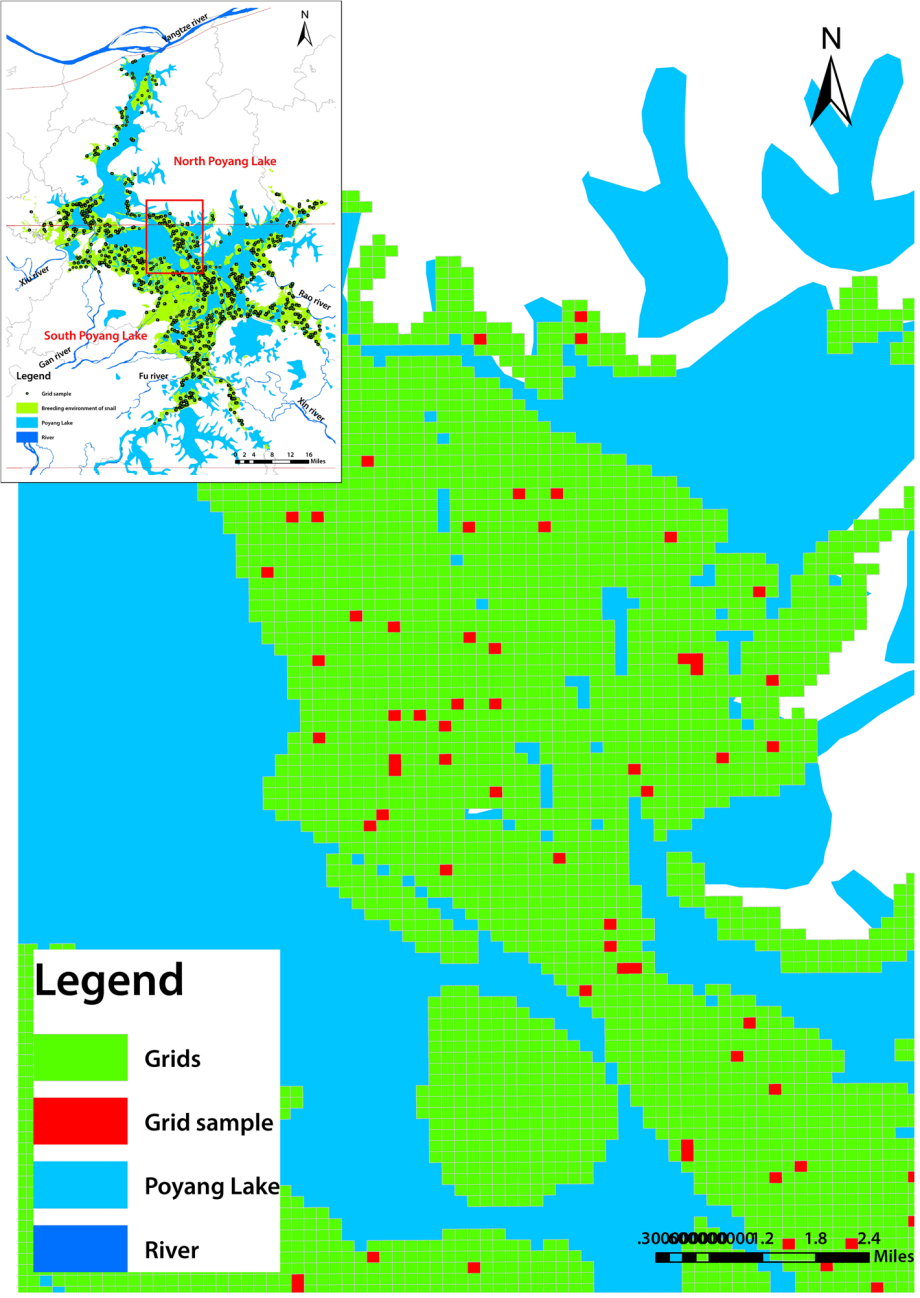
Geographical spatial map of the 200  × 200 m grids and sampling grid for snail survey (local magnified). The green squares represent vector girds created by ArcGIS software. The red ones represent sampling grids for snail survey

There were a total 62 340 grids created for the marshlands of Poyang Lake, of which, 37 754 grids were selected as a valid sample when its area was above 24 000 m^2^ in more than half the area of each grid. There are 31 207 in the south sector and 6547 valid grids in the north sector of Poyang Lake, respectively. 1159 grids were randomly selected for the *Oncomelania* snail survey, accounting for 3.07% of the total valid grids, with 949 grids in the south and 210 grids in the north (Figs. 1 and 2).

### Field survey

The field survey was conducted in 2015 and 2016. Each selected grid was surveyed for snails with taking one side of each grid as the starting line. In each grid one survey frame of 0.1 m^2^ was set up within a 50 m × 50 m area. There were 25 spots in each grid that were surveyed. However, some spots were submerged in water but no less than ten spots were obtained. All snails in each frame were collected and the GPS coordinates of each frame were recorded. The collect snails were placed on a glass plate, covered by another glass plate, and the shells of snails were crushed slowly. A drop of water was added to each crushed snail. Those who had fresh soft tissue with a contractile response were considered living snails. The plate was then placed under a microscope or dissection microscope in order to identify a possible infection of *Schistosoma japonicum*.

### Processing of elevation data

Based on the topographic map of Poyang Lake bottom in 2009, the Digital Elevation Model (DEM) grids of the topographic map were projected into the WGS-84 coordinate system and Transverse Mercator projection using the ArcGIS software. The DEM data is preprocessed before interpolation, which includes separating the conglutinated contour line and connecting the disconnected contour line, establishing the elevation index table at the same time, and then calculating the elevation data interpolation. The method of contour raster image profile interpolation was used for elevation data interpolation in Contour Line rendering. The elevation accuracy was evaluated by the checkpoint method and contour playback methodolgy. The elevation data corresponding to the spatial position of each surveyed snail frame was extracted by using the software tool of “Extract Values to Points”.

### Data analysis

All snail survey data were collected and statistically analyzed with SPSS 20.0 software (IBM, Armonk, USA). Two statistical indicators, percent of frames with living snails and mean density of living snails, were analyzed. Chi-squared test (*χ*^2^) test was used to determine the percent of frames with living snail with *P* < 0.05 considered as statistically significant. Considering the negative binomial distribution of *Oncomelania hupensis* snails and the number of snails in most survey frame being zero, a logarithmic variance analysis was used to analyze the mean density of living snail. Firstly, the number of snails in each survey frame plus one and the logarithm was taken. Then, the logarithmic arithmetic mean was calculated. After that, antilog minus one was taken. Finally, variance analysis was used to determine the mean density of living snail with *P* < 0.05 considered as statistically significant. The formula for calculating the two indicators is as following:

$$ \mathrm{percent}\kern0.17em \mathrm{of}\kern0.17em \mathrm{frames}\kern0.17em \mathrm{with}\kern0.17em \mathrm{living}\kern0.17em \mathrm{snail}\;\left(\%\right)=\frac{\mathrm{frames}\kern0.17em \mathrm{of}\kern0.17em \mathrm{living}\kern0.17em \mathrm{snail}}{\mathrm{Number}\kern0.17em \mathrm{of}\kern0.17em \mathrm{investigated}\kern0.17em \mathrm{frames}}x100\% $$

$$ \mathrm{mean}\kern0.17em \mathrm{density}\kern0.17em \mathrm{of}\kern0.17em \mathrm{living}\kern0.17em \mathrm{snail}\left(\mathrm{No}./0.1{\mathrm{m}}^2\right)={\lg}^{\hbox{-} 1}\Big(\frac{\sum \lg\;\left(\mathrm{number}\kern0.17em \mathrm{of}\kern0.17em \mathrm{living}\kern0.17em \mathrm{snail}+1\right)}{\mathrm{Number}\kern0.17em \mathrm{of}\kern0.17em \mathrm{investigated}\kern0.17em \mathrm{frames}}-1 $$

The snail frame elevation was graded at 1 meter intervals, then, the percent of frames with living snails and the mean density of living snails at each elevation grade were calculated in the southern and northern sectors of the lake to determine snail-concentrated elevation intervals.

## Results

### Snail survey

**Fig. 3:**
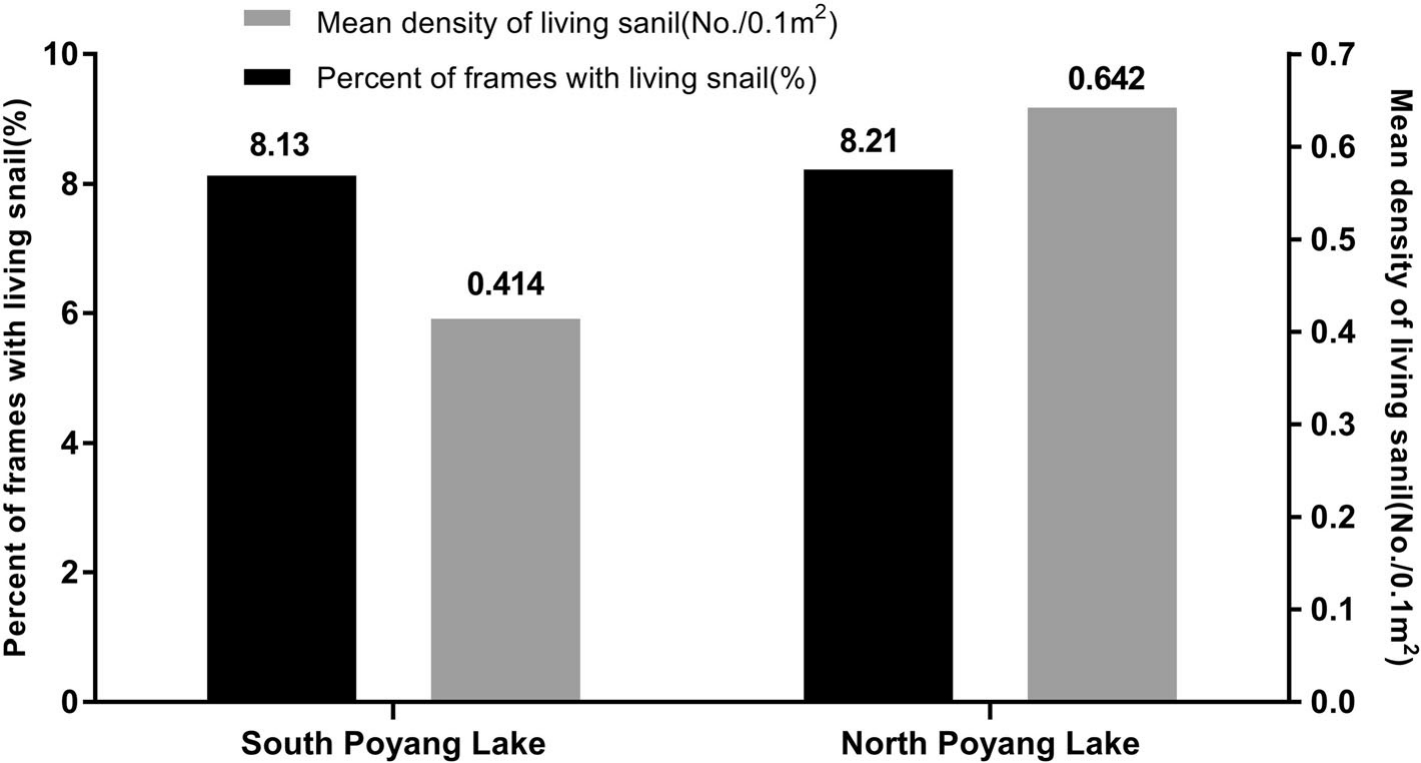
Investigation of *Oncomelania hupensis* snails in different areas of Poyang Lake

A total of 1159 potential snail sampling grids were surveyed, of which 15 231 frames were investigated. 1241 frames had live *Oncomelania* snails corresponding to 8.15% of the total number of frames. The mean density of live snails was 0.141/0.1m^2^, with a maximum of 57 snails per frame (0.1 m^2^). The percent of frames with snails in the southern sector (8.13%) of Poyang Lake did not differ statistically from the north (8.21%). However, the mean density of live snails in the northern sector (0.164/0.1m^2^) of the lake was statistically higher (*F* = 6.73; *P* = 0.01) than the south (0.141/0.1m^2^) (Fig. 3).

### Elevation of survey frames

The data to determine elevation of each survey frame was extracted using a topographic map of the Poyang Lake bottom and was examined for abnormal values using the Box Diagram Method. There were a total 94 abnormal elevation values that were discarded from further statistical analysis. The frame elevation ranged from 10-16 m, accounting for 97.7% of the total number of frames. The highest and lowest elevations were 19.7 m and 5 m, respectively. The elevation of surveyed frames in the southern lake and the northern lake ranged from 9.0 - 19.7 m and 5.0–19.7 m, respectively (Table 1).

**Table 1:** Elevation of survey frames in the Poyang Lake region of China

Elevation grading (m)	South Poyang Lake				North Poyang Lake				Total			
	Frequency	Composition ratio (%)	Max. (m)	Min. (m)	Frequency	Composition ratio (%)	Max. (m)	Min. (m)	Frequency	Composition ratio (%)	Max. (m)	Min. (m)
5.0–5.9					10	0.31	5.9	5.0	10	0.07	5.9	5.0
6.0–6.9					3	0.09	6.0	6.0	3	0.02	6.0	6.0
7.0–7.9					3	0.09	7.9	7.0	3	0.02	7.9	7.0
8.0–8.9					22	0.68	8.8	8.1	22	0.15	8.8	8.1
9.0–9.9	19	0.16	9.8	9.0	147	4.58	9.8	9.0	166	1.10	9.8	9.0
10.0–10.9	254	2.13	10.8	10.0	619	19.27	10.8	10.0	873	5.77	10.8	10.0
11.0–11.9	2030	17.02	11.9	11.0	1048	32.63	11.8	11.0	3078	20.33	11.9	11.0
12.0–12.9	3542	29.70	12.9	12.0	747	23.26	12.8	12.0	4289	28.33	12.9	12.0
13.0–13.9	2685	22.52	13.9	13.0	329	10.24	13.8	13.0	3014	19.91	13.9	13.0
14.0–14.9	2617	21.95	14.9	14.0	216	6.72	14.8	14.0	2833	18.72	14.9	14.0
15.0–15.9	660	5.53	15.8	15.0	42	1.31	15.8	15.0	702	4.64	15.8	15.0
16.0–16.9	69	0.58	16.8	16.0	14	0.44	16.7	16.0	83	0.55	16.8	16.0
17.0–17.9	28	0.23	17.8	17.0	5	0.16	17.7	17.0	33	0.22	17.8	17.0
18.0–18.9	20	0.17	18.8	18.0	5	0.16	18.7	18.0	25	0.17	18.8	18.0
19.0–19.9	1	0.01	19.7	19.7	2	0.06	19.7	19.1	3	0.02	19.7	19.1
合计	11 925	100.00	19.7	9.0	3212	100.00	19.7	5.0	15 137	100.00	19.7	5.0

*Max.* Maximum, *Min.* Minimum

### Elevation of frames with Oncomelania snails

The elevation of snail frames ranged from 11.1 m to 15.8 m in the southern sector of the lake and from 9.3 m to 15.6 m in the north sector of the lake. As shown below in Table 2, the mean elevation of surveyed *Oncomelania* frames in the southern sector (13.2 m) of the lake was significantly higher (*F* = 215.2; *P* < 0.01) than in the northern sector (12.0 m).

**Table 2:** Elevation of *Oncomelania* surveyed frames in the Poyang Lake region of China

Area	Number of survey point	Elevation (m)		
		Mean	Median	Extremum
South Poyang Lake	973	13.2 ± 1.1	12.9	11.1–15.8
North Poyang Lake	263	12.0 ± 1.2	12.0	9.3–15.6

### Snail-concentrated elevation intervals

In the southern sector of the lake, the elevation with the highest percentage of living snails was at 15–16 m (18.2%), and secondly at 12–13 m (12.1%). In the north sector of the lake, the elevation range with highest percentage of living snails was at 15–16 m (14.3%), and secondly at 12–14 m (11.5%). The mean density of living snails (0.387/0.1m^2^) at an elevation of 15–16 m in the southern lake was significantly higher (*F* = 129.6; *P* < 0.01) than that of other elevations recorded in the sector. In the northern lake region, the mean density of living snails at an elevation of 12–14 m was 0.239/0.1m^2^. This was again significantly higher (*F* = 20.8; *P* < 0.01) than the other elevations recorded (Table 3).

**Table 3:** Snail-concentrated elevation intervals in the Poyang Lake region of China

Elevation grading (m)	South Poyang Lake		North Poyang Lake	
	Percent of frames with living snail (%)	Mean density of living snail (No./0.1m^2^)	Percent of frames with living snail (%)	Mean density of living snail (No./0.1m^2^)
< 9.0	–	–	0	0
9.0–9.9	0	0	3.40	0.079
10.0–10.9	0	0	8.72	0.185
11.0–11.9	3.79	0.056	6.87	0.116
12.0–12.9	12.06	0.205	10.31	0.229
13.0–13.9	7.49	0.125	11.55	0.263
14.0–14.9	5.62	0.085	5.09	0.098
15.0–15.9	18.18	0.387	14.29	0.178
> 16.0	0	0	0	0

## Discussion

The vast marshlands of the Poyang Lake region are historically an endemic zone for zoonotic schistosomiasis in Jiangxi province. The marshlands surrounding Poyang Lake are distributed over 13 counties. In the mid-1980s, based on elevations provided by the hydrological stations in the Poyang Lake region, maximum and minimum elevations of 615 marshlands were calculated and *Oncomelania* snails surveyed. The survey indicated that snails were distributed at ‘three marshland belts’ at an elevation 11–16 m. The upper belt had few snail-infested areas, the middle belt was deemed snail-concentrated, while lower belt, like the upper belt, had few snails. Thus, 95% of the *Oncomelania* snail habitat in the lake region was distributed in marshlands at elevation ranging from 12 to 15 m. This snail-concentrated belt is typically submerged during the flood season from April to May, and reemerges after the water reced in October or November. There were virtually no snails (< 3%) in the marshlands at elevations below 11 m or at elevations greater than 16 m [25].

Snail distribution fluctuates with the water level. Water levels that are too high (flooding) or too low (drought) will affect the reproduction of snails [26]. In a study in 2013, Feng et al. reported that the average marshland area in Poyang Lake region increased significantly by 385.7 km^2^ in 2004–2009 (after the closure of the Three Gorges Dam) as compared with that in 2000–2002 (before dam closure) [27]. A comparison of water levels in Poyang lake between 1993 and 2012 found that the proportion of very low water level (< 10 m; Wusong elevation) after 2003 had soared from 4.34 to 24.8%, and the dry season after 2003 had lasted longer [28]. Thus, snail reproduction would have certainly been impacted. It was also observed that at this water level (< 10 m) there was a change in snail distribution with more suitable snail environments situated further of north [29].

In this study, we investigated the distribution of *Oncomelania* snails in the Poyang Lake region using both GIS and GPS in order to ascertain the dynamic changes the snail distribution pattern at different elevations in the marshlands and to further define the snail habitat. A total of 1159 potential snail sampling grids were surveyed, of which 15 231 frames (0.1 m^2^/frame) were investigated. 7049 frames had live *Oncomelania* snails corresponding to 8.15% of the total number of frames sampled. The mean density of living snails was 0.463/0.1 m^2^. The percent of frames with snails in the southern sector (8.13%) of the Poyang Lake did not differ statistically from the north (8.21%). However, the mean density of live snails in the northern sector (0.164/0.1 m^2^) of the lake was statistically greater than the south (0.141/0.1 m^2^). This finding was similar to that reported by Hu et al [29].

Historical data has shown that the elevation of snail-infested marshlands in Poyang Lake is at 10–16 m, and predominately between 12 and 14 m [30]. The present distribution of snails in southern and northern sectors of Poyang Lake has changed significantly, and the snail-concentrated belts have also changed correspondingly. In the south sector of the lake, the snail-inhabited marshlands is distributed at 11–16 m and were divided two snail-concentrated belts (e.g., 12–13 m and 15–16 m of elevation). In the north sector of the lake, the snail-inhabited marshlands ranged from 9 to 16 m in elevation, with snail-concentrated belt at 12–14 m. There are two possible reasons that may explain the difference in the density of snails between the south and north of Poyang Lake. Firstly, the elevations of all marshlands in the south of Poyang Lake are obviously higher than those in the north. In recent years, the dry season in autumn and winter in the lake advanced and extended. As a result the marshlands with high altitude were not flooded or rarely flooded, which had a negative effect on egg laying and hatching of snails. As a consequence the density of snails in the south was greatly reduced. In contrast, dynamic changes in the water level had less impact on the marshlands in the middle and low altitudes in the north of the lake. These marshlands are typically flooded in the spring, autumn and winter and are thus more suitable for snail breeding and reproduction, which lead to the increase or higher density of the snails. Secondly, with a sustained lower water level, the soil moisture content in the high elevation marshlands in the south of the lake were reduced, making egg laying and hatching of snails difficult, which ultimately resulted in a decrease of snail density. At the same time, the low elevation swamp lands in the north of the lake has now evolved into grasslands, resulting in the increase of marshland area and the migration of snails down to the waterline. Consequently, both the area of snail-infested marshlands and snail density in the north of the lake increased. The lower snail-infested elevation of marshland in the southern lake was two meters higher than that in the northern lake, which indicated an eco-environment suitable for snail survival and that breeding was moving from the southern lake to lower elevated areas of the northern lake. Whether this trend of snail distribution in the marshland of Poyang Lake will continue and is caused by the Three Gorge Dam is yet to be fully determined. Previous studies have confirmed that the distribution of snail in the lake region is closely related to vegetation species in addition to elevation [31]. The relationship between the current snail distribution and vegetation needs further study in the future.

## Conclusions

*Oncomelania hupensis*, the snail intermediate host of schistosomiasis in China, is distributed in the marshlands of Poyang Lake at an elevation of 9–16 m elevation and the distribution of snails has changed over time due to changes in the water level post closure of the Three Gorges Dam. The outcomes are important for national integrated schistosomiasis control strategies leading to disease elimination. Based on the current geological features of the snail habitant, focused mollusciciding should occur in northern regions within identified snail hotspots of transmission.

## Additional file

Additional file 1: Multilingual abstracts in the five official working languages of the United Nations. (PDF 694 kb)

## References

[1] Zhou XN, Wang LY, Chen MG, Wu XH, Jiang QW, Chen XY (2005). The public health significance and control of schistosomiasis in China--then and now. Acta Trop.

[2] Zou L, Ruan SG (2015). Schistosomiasis transmission and control in China. Acta Trop.

[3] Zhou XN (2016). Implementation of precision control to achieve the goal of schistosomiasis elimination in China. Zhongguo Xue Xi Chong Bing Fang Zhi Za Zhi.

[4] Zhang LJ, Xu ZM, Qian YJ, Dang H, Lv S, Xu J (2017). Endemic status of schistosomiasis in People’s republic of China in 2015. Zhongguo Xue Xi Chong Bing Fang Zhi Za Zhi..

[5] Liu L, Yang GJ, Zhu HR, Lin A (2014). Knowledge of, attitudes towards, and practice relating to schistosomiasis in two subtypes of a mountainous region of the People’s republic of China. Infect Dis Poverty.

[6] Hu Y, Xiong CL, Zhang ZJ, Luo C, Ward M, Gao J (2014). Dynamics of spatial clustering of schistosomiasis in the Yangtze River valley at the end of and following the World Bank loan project. Parasitol Int.

[7] Tseng KH, Liang S, Ibaraki M, Lee H, Shum CK (2014). Study of the variation of schistosomiasis risk in Lake Poyang in the People’s republic of China using multiple space-borne sensors for monitoring and modelling. Geospat Health.

[8] Li YS, Raso G, Zhao ZY, He YK, Ellis MK, McManus DP (2007). Large water management projects and schistosomiasis control, Dongting Lake region, China. Emerg Infect Dis.

[9] Hao Y, Wu XH, Zhen H, Wang LY, Guo JG, Xia G (2007). Schistosomiasis situation in People’s Republic of China in 2006. Zhongguo Xue Xi Chong Bing Fang Zhi Za Zhi..

[10] Liu YB, Wu GP, Zhao XS (2013). Recent declines in China’s largest freshwater lake: trend or regime shift?. Environ Res Lett.

[11] Du YL, Zhou HD, Peng WQ, Liu ZB, Wang SY, Yin SH (2015). Modeling the impacts of the change of river-Lake relationship on the hydrodynamic and water quality revolution in Poyang Lake. Acta Sci Circumstant.

[12] Liu XD, Ren BF (2014). Analysis on variation characteristics and genesis of lower water level of Poyang Lake. Yangtze River.

[13] Chen HG, Zeng XJ, Lin DD, Lv SB, Gu XN, Hang CQ (2013). The changes of hydrological regime in Poyang Lake after runs of three gorges project and its impact on prevalence of schistosomiasis in the lake region. Zhongguo Xue Xi Chong Bing Fang Zhi Za Zhi..

[14] Wang XY, He J, Yang K, Liang S (2016). Applications of spatial technology in schistosomiasis control programme in the People’s Republic of China. Adv Parasitol.

[15] Li FY, Ma SJ, Li YY, Tan HZ, Hou XY, Ren GH (2017). Impact of the three gorges project on ecological environment changes and snail distribution in Dongting Lake area. PLoS Negl Trop Dis.

[16] Zhu HR, Liu L, Zhou XN, Yang GJ (2015). Ecological model to predict potential habitats of *Oncomelania hupensis*, the intermediate host of *Schistosoma japonicum* in the mountainous regions, China. PLoS Negl Trop Dis.

[17] Wang Y, Zhuang DF (2015). A rapid monitoring and evaluation method of schistosomiasis based on spatial information technology. Int J Environ Res Public Health.

[18] Wei ZH, Li YK, Xu P, Qian FW, Shan JH, Tu XB (2016). Patterns of change in the population and spatial distribution of oriental white storks (*Ciconia boyciana*) wintering in Poyang Lake. Zool Res.

[19] Zhang ZX, Chen X, Xu CY, Hong Y, Hardy J, Sun ZH (2015). Examining the influence of river-Lake interaction on the drought and water resources in the Poyang Lake basin. J Hydrol.

[20] Feng L, Hu C, Chen X (2012). Satellites capture the drought severity around China’s largest freshwater lake. IEEE J STARS.

[21] Hong F, Chen WJ, Zhou HM, Chen JS (2010). Discussion on impact of Poyang Lake ecological water control project on aquatic biology. Jiangxi Sci.

[22] Lu JY, Yao SM, Shao XJ, Zhang XB (2012). Response process of the lower reaches of the three gorges dam after the initial operation.

[23] Hu F, Lin DD, Yuan M, Liu YM, Li ZJ, Li JY (2014). Study on the digital mapping of *Oncomelania* snails in marshland of Poyang Lake region based on GIS. Jiangxi Med J.

[24] Chen Z, Gu XN, Lv SB, Li YF, Jiang WS, Hang CQ (2015). Endemic situation of schistosomiasis in the national surveillance sites in Jiangxi Province from 2005 to 2014. J Trop Dis Parasitol.

[25] Zhang SJ, Liu ZD, Li GH, Zhong JH, Chen Y (1988). Study on snail condition investigation and snail control measures in Poyang Lake area. Yi Xue Yan Jiu Tong Xun.

[26] Chen HG, Lin DD (2004). The prevalence and control of schistosomiasis in Poyang Lake region, China. Parasitol Int.

[27] Feng L, Hu C, Chen X, Zhao X (2013). Dramatic inundation changes of China’s two largest freshwater lakes linked to the three gorges dam. Environ Sci Technol.

[28] Mei XF, Dai ZJ, Du JZ, Chen JY (2015). Linkage between three gorges dam impacts and the dramatic recessions in China’s largest freshwater Lake, Poyang Lake. Sci Rep.

[29] Hu F, Liu YM, Li ZJ, Yuan M (2012). Effect of environmental factors on temporal and spatial distribution of schistosomiasis in Poyang Lake region. Zhongguo Xue Xi Chong Bing Fang Zhi Za Zhi.

[30] Zhang B (1988). Studies on Poyang lake.

[31] Li ZJ, Chen HG, Gong P, Zeng XJ, Liu YM, Xie SY (2010). Study on relationship between vegetation and spatial distribution of *Oncomalania* snail in Poyang Lake region. Zhongguo Xue Xi Chong Bing Fang Zhi Za Zhi..

