# Supplementary material for: Distribution pattern of the snail intermediate host of schistosomiasis japonica in the Poyang Lake region of China
Source: Infect Dis Poverty. 2019 Mar 29;8:23. doi: 10.1186/s40249-019-0534-8 (PMC6440081; doi:10.1186/s40249-019-0534-8)
Supplement: Supplementary file 1 — Multilingual abstracts in the five official working languages of the United Nations. (PDF 694 kb) [file 40249_2019_534_MOESM1_ESM.pdf]

Translation of the abstract into the five official working languages of the United Nations

نموذج توزيع العائل الوسيط لقوقع البلهارسيا اليابانية في منطقة بحيرة بويانغ الصينية

Sheng Li, Allen G. Ross -Ming Liu, Yue-Jun Li, Min Yuan, Zhe Chen, Yue-Feng Li, Zhao-Biao Lv, Yi-Fei Hu, Jun Ge, Shang Dan Lin-and Dan

ملخّص

معلومات تأسيسية : مع إغلاق سد الممرات الثلاثة سنة 2003، تغيرت الطبيعة المائية لبحيرة البويانغ تغيرًا هائلاً أدى إلى تغيرات بيئية جوهرية. ومن أجل تقييم أثر ذلك على البلهارسيا، قيّمت الدراسة هاهنا الأنماط الزمانية والمكانية للعائل الوسيط لقوقع Oncomelaniahupensis في روافد بحيرة البويانغ. وتتضمن استنتاجات الدراسة على توابع ضمنية هامة بشأن الاستراتيجيات المستقبلية للسيطرة على القوقع ممى يؤدي إلى القضاء على المرض..

الأساليب المستخدمة: قُسّمت منطقة الأهوار المحيطة ببحيرة البويانغ عشوائيًا إلى شبكات اتجاهية مساحة كل منها 200 × 200 متر وذلك باستخدام برنامج ArcGIS، واختار البرنامج الشبكات الخاضعة للفحص عشوائيًا. أُجري فحص القواقع في كل شبكة مُختارة باستخدام إطار فحص مساحته 50 × 50 متر مع اتخاذ خط جانبي في كل شبكة ليكون خط البداية. وقد استُخدِم أكثر من عشرة أطر لكل شبكة خاضعة للفحص مع تسجيل الموقع الجغرافي لكلٍ منها على النظام العالمي لتحديد المواقع. وجُمِعت القواقع الموجودة في كل إطار كلها من أجل تحديد حالة العدوى باستخدام المجهر. واستُخرِجت بيانات ارتفاع جميع الأطر من خريطة طوبوغرافية لقاع البحيرة من أجل تحليل متوسط الارتفاع. كذلك جُمِعت جميع بيانات فحص القواقع وحُلِلت إحصائيًا باستخدام برنامج SPSS 20.0 من أجل تحديد النسب المختلفة في الأطر التي تحتوي على قواقع والكثافة الوسطى وجُمِعت جميع بيانات فحص القواقع وحُلِلت إحصائيًا باستخدام برنامج SPSS 20.0 من أجل تحديد النسب المختلفة في الأطر التي تحتوي على قواقع والكثافة الوسطى للقواقع الحية في أقاليم مختلفة من بحيرة البويانغ. وبعد ذلك حُدِدت ارتفاعات الأهوار الموبوءة بالقواقع وأوكار هذه القواقع.

النتائج: تم فحص إجمالي 1159 شبكة يحتمل وجود قواقع بها، وكان من بينها 15 231 إطار (0,1 متر^{2}/إطار)تم تحليلها. ووُجِد في 1241 إطار قواقع Oncomelania حية أي ما يعادل 8,15%من إجمالي عدد الأطر. وقُدرت الكثافة الوسطى للقواقع الحية بمقدار 0,1/0,463 متر^{2} بحد أقصى 57 قوقع للإطار. ولم تختلف نسبة الأطر التي بها قواقع في القطاع الجنوبي للبحيرة والتي تُقدر ب (8,13%) إحصائيًا عن نسبة الإطر في القطاع الشمالي والمقدرة ب (8,21%). إلا أن وسط كثافة القواقع الحية في القطاع الشمالي والمقدر ب(0,1/0,164 متر^{2}) من البحيرة كان أعلى إحصائيًا من (F =6.727; P = 0.010) أعلى من الجنوب المقدر ب(0,1/0,141 متر^{2}). وفي جنوب البحيرة تراوح ارتفاع الأهوار الموبوءة بالقواقع من 11 إلى 16 متر ويمكن تقسيمه إلى حزامين لتركيز القواقع تتراوح ارتفاعاتهما بين ارتفاع 23 إلى 13 متر وارتفاع 15 إلى 16 متر على التوالي. أما في شمال البحيرة، فتراوح ارتفاع الأهوار الموبوءة بالقواقع من 9 إلى 16 متر بينما كان ارتفاع المنطقة المتركز بها القواقع من 12 إلى 14 متر.

الاستنتاجات: تراوح ارتفاع الأهوار الموبوءة بالقواقع في منطقة بحيرة البويانغ من 9 إلى 16 متر، وانتقلت مواطن توزيع القواقع إلى الشمال وإلى ارتفاع أقل بسبب تغير مستوى الماءبعد إغلاق السد. ووفقًا للسمات الجيولوجية الحالية لمناطق سكنى القواقع فيجب تطبيق المبيدات الرخوة على المناطق الشمالية الكثيفة بالقواقع والتي يتردد عليها البقر والبشر دائمًا. إذ أن استهداف هذه المناطق المتعارف عليها بأنها "بقع ساخنة" لانتقال القواقع من شأنه أن يساعد في جهود القضاء عليها.

Translated from English version into Arabic by Mohamed Aglan, proofread by Mohamed Taha Mahmoud, through

TRANSLATORS
WITHOUT BORDERS

中国鄱阳湖区钉螺分布态势研究

胡飞，葛军，吕尚标，李宜峰，李召军，袁敏，陈喆，刘跃民，李岳生，Allen G. Ross，林丹丹

摘 要

引言：随着 2003 年长江三峡水利枢纽工程运行，鄱阳湖水文环境发生明显改变。为评估鄱阳湖水文环境变化对血吸虫病的影响，本研究调查分析了湖区钉螺的分布态势，以期为鄱阳湖区域制定钉螺控制规划，进而消除血吸病提供科学依据。

方法：借助 ArcGIS 软件对鄱阳湖区草洲空间数据按 200 m × 200 m 创造矢量网格，对网格进行随机抽样，并对抽取的网格以 50 m × 50 m 设框开展系统抽样法调查螺情，每个网络样本内调查框不少于 10 个框，并利用 GPS 仪记录调查框的空间地理信息；解剖每个调查框内检获的钉螺以了解感染情况；用 SPSS20.0 分析鄱阳湖不同区域活螺框数和平均活螺密度的差异及变化，同时以鄱阳湖区湖底地形图为基础提取所有调查框的高程数据，分析有螺调查框的平均高程和极值，明确钉螺在草洲分布高程范围以及鄱阳湖区密螺带区域。

结果：共调查 1159 个有效网格，15 231 个调查框（每框面积为 0.1 m^2），活螺框出现率为 8.15%（1241/15 231），活螺平均密度为 0.463 只/0.1 m^2，每框最高查获钉螺 57 只，其中鄱阳湖南区和北区有螺框出现率分别为 8.13%和 8.21%，两者间差异无统计学意义，而鄱阳湖北区活螺平均密度(0.642 只/0.1 m^2)显著高于南区（0.414 只/0.1 m^2）（F = 6.727; P = 0.010）。鄱阳湖南区有螺高程为 11−16 m，可分为 12−14 m 和 15−16 m 两个密螺带；鄱阳湖北区有螺高程为 9−16

m，密螺带区域高程为 12−14 m。
结论：鄱阳湖区草洲钉螺分布在 9−16 m 的高程范围内，但由于三峡大坝运行后水位的影响，鄱阳湖不同区域钉螺分布特征不同，钉螺繁殖分布生境向鄱阳湖北部及下高程区域移动。基于当前钉螺分布特征，鄱阳湖北区人畜常到的区域应是灭螺的重点地区。针对明确的血吸虫病传播的“热点”采取干预措施，将推动鄱阳湖区的血吸虫病消除进程。

Translated from English version into Chinese by Jun Ge

Schéma de répartition des escargots servant d’hôtes intermédiaires au Schistosoma japonica dans la région du lac Poyang, en Chine

Fei Hu, Jun Ge, Shang-Biao Lv, Yi-Feng Li, Zhao-Jun Li, Min Yuan, Zhe Chen, Yue-Ming Liu, Yue-Sheng Li, Allen G. Ross et Dan-Dan Lin

Résumé
Contexte: l’achèvement du barrage des Trois Gorges en 2003 a profondément modifié l’hydrologie du lac Poyang est son environnement. Afin d’évaluer l’impact de ces changements sur la schistosomiase, la présente étude a évalué les schémas de répartition dans le temps et l’espace de son hôte intermédiaire, l’escargot *Oncomelania hupensis*, dans les ruisseaux tributaires du lac Poyang. Les résultats de l’étude ont des implications importantes sur l’élaboration de stratégies de contrôle des gastéropodes en vue de l’élimination de la maladie.
Méthodes: les terres marécageuses environnant le lac Poyang ont été divisées de façon aléatoire selon un maillage de 200 × 200 m à l’aide du logiciel ArcGIS et les carrés étudiés ont été sélectionnés de façon aléatoire par le logiciel. Le recensement des escargots a été réalisé dans chaque carré sélectionné, avec un maillage de 50 × 50 m partant d’un côté de chaque carré. Nous avons utilisé au moins dix subdivisions de chaque carré étudié, en enregistrant les coordonnées GPS de chacune. Tous les escargots présents dans chaque subdivision ont été récoltés afin de déterminer leur statut d’infestation par examen au microscope. Les données d’altitude de toutes les subdivisions ont été extraites d’une carte topographique du fond du lac afin d’analyser l’altitude moyenne. Toutes les données de recensement des escargots ont été recueillies et ont fait l’objet d’une analyse statistique avec le logiciel SPSS 20.0, afin de déterminer la différence de pourcentage de carrés contenant des escargots vivants et de densité moyenne d’escargots vivants entre différentes zones du lac Poyang. L’altitude des marécages infestés de gastéropodes et des concentrations d’escargots ont ensuite été identifiés.
Résultats: au total, 1159 carrés de prélèvement d’escargots ont été étudiés et 15 231 subdivisions (0,1 m^2/subdivision) y ont été explorées. Nous avons trouvé des exemplaires vivants d’*Oncomelania* dans 1241 subdivisions, soit 8,15 % du total. La densité moyenne d’escargots vivants était de 0,463/0,1 m^2 et on a atteint un maximum de 57 individus par subdivision. Le pourcentage de subdivisions contenant des escargots dans le secteur sud du lac Poyang (8,13 %) n’était pas significativement différent de celui du secteur nord (8,21 %). La densité moyenne d’escargots vivants dans le secteur nord du lac (0,164/0,1 m^2) était cependant significativement (F = 6,727 ; P = 0,010) plus grande que dans le secteur sud (0,141/0,1 m^2). Dans le sud du lac, l’altitude des habitats des escargots était comprise entre 11 et 16 m et on pourrait encore la subdiviser en deux bandes de concentration à 12-13 m et 15-16 m. Dans le nord du lac, l’altitude de la zone marécageuse habitée par les escargots s’étendait entre 9 et 16 m, avec une concentration entre 12 et 14 m.
Conclusions: l’altitude des marécages infestés d’escargots autour du lac Poyang était de 9 à 16 mètres. La distribution et l’habitat des gastéropodes se sont décalés vers le nord du lac et à une altitude plus basse à la suite des changements du niveau de l’eau produits par la fermeture du barrage. D’après les caractéristiques géologiques de l’habitat des gastéropodes, un épandage ciblé de molluscicides devrait être effectué dans les régions du nord du lac, où l’infestation par les escargots est dense et les passages de bovins et d’humains fréquents. Le traitement ciblé de ces « foyers » de transmission identifiés aidera à lutter contre la schistosomiase.

Translated from English version into French by Suzanne Assenat, proofread by Eric Ragu, through

TRANSLATORS
WITHOUT BORDERS

Модель распространения улитки, промежуточного хозяина японского шистосоматоза, в районе озера Поянху в Китае

Fei Hu, Jun Ge, Shang-Biao Lv, Yi-Feng Li, Zhao-Jun Li, Min Yuan, Zhe Chen, Yue-Ming Liu, Yue-Sheng Li, Allen G. Ross and Dan-Dan Lin (Фей Ху, Юн Ге, Шанг-Бьяо Лв, Йи-Фенг Ли, Жао-Юн Ли, Мин Юань, Же Чен, Юэ-Минг Лиу, Юэ-Шенг Ли, Аллен Росс и Дан-Дан Лин)

Аннотация

Справка: С закрытием элеткростанции Три Ущелья в 2003 году гидрология озера Поянху сильно изменилась, вызвав значительные изменения в окружающей среде. Чтобы определить, как это повлияло на распространение шистосоматоза в притоках озера Поянху, в данном исследовании были проанализированы пространственные и временные модели распространения улитки, *Oncomelania hupensis*, являющейся его промежуточным хозяином. Результаты этого исследования имеют важное значение для будущих стратегий по контролю популяции улиток, что может привести к устранению болезни.

Методы: Заболоченная территория вокруг озера Поянху была случайным образом расчерчена на векторные сектора 200 × 200 метров (м) с использованием программного обеспечения ArcGIS. Исследуемые сектора были отобраны данной программой также случайным образом. Исследование улиток проходило в каждом выбраном секторе с использованием участка размером 50 × 50 м, где боковая линия каждого сектора служила начальной. Не менее 10 участков использовались в каждом секторе и записывались через глобальную систему определения координат GPS. На каждом участке собирались все улитки, для того чтобы с помощью микроскопии определить их инфекционный статус. Данные о высоте каждого участка были взяты с топографической карты дна озера, для того чтобы проанализировать среднестатистическую высоту. Все данные исследования об улитках были собраны и статистически проанализированы с помощью программного обеспечения SPSS 20.0. Цель анализа состояла в определении разницы процентного соотношениия между участками с живущими на них улитками и средней плотностью проживающих улиток в других районах озера Поянху. Последовательно определены были как высота заболоченных территорий, населенных улитками, так и их места обитания.

Результаты: В общей сложности было рассмотрено 1159 секторов с потенциальной выборкой улиток, из которых исследован 15231 участок (0,1 м^2/участок). На 1241 участке проживали улитки *Oncomelania*, что соответствует 8,15% от общего количества участков. Средняя плотность обитаемых улиток составляла 0,463/0,1 м^2 с максимальным количеством в 57 улиток на участок. Процент участков с улитками в южной части озера Поянху (8,13%) статистически не отличался от северной (8,21%). Однако средняя плотность обитания улиток в северной части озера (0,164/0,1 м^2), была статистически большей (F =6,727; P = 0,010), чем в южной (0,141/0,1 м^2). На юге озера высота заболоченной территории обитания улиток варьировалась в пределах 11-16 м. Ее в дальнейшем можно бы было разбить на два пояса с соответственным сосредоточением улиток на уровне 12-13 м и 15-16 м. На севере озера высота заболоченной территории обитания улиток варьировалась в пределах 9-16 м, где зона наибольшей их концентрации приходилась на высоту в 12-14 м.

Вывод: Высота заболоченных территорий, заселенных улитками в районе озера Поянху, варьируется в пределах 9-16 м. Распределение улиток и ареал обитания переместились к северу озера, а также на меньшие высоты из-за изменения уровня воды после закрытия электростанции. Основываясь на актуальных геологических данных, направленное уничтожение моллюскоцидами ареала обитания улиток должно осуществляться в северных районах с их плотной популяцией, где также часто проходят люди и крупный рогатый скот. Целеустремленное воздействие на такие локализированные "горячие точки" передачи болезни поможет с инициативой ее преодоления.

Translated from English version into Russian by Anastasiia Dobrosynets, proofread by Dariia Moss, through

TRANSLATORS
WITHOUT BORDERS

Patrón de distribución del caracol huésped intermediario de esquistosomiasis japonica en la región china del lago Poyang

Fei Hu, Jun Ge, Shang-Biao Lv, Yi-Feng Li, Zhao-Jun Li, Min Yuan, Zhe Chen, Yue-Ming Liu, Yue-Sheng Li, Allen G. Ross y Dan-Dan Lin

Resumen

Contexto: con el cierre de la presa de las Tres Gargantas en 2003, la hidrología del lago Poyang cambió drásticamente, dando

lugar a cambios significativos en el entorno. Para evaluar el impacto de la esquistosomiasis, este estudio examinó los patrones espaciales y temporales del caracol huésped intermediario, *Oncomelania hupensis,* en los afluentes del lago Poyang. Los resultados del estudio tienen importantes consecuencias para futuras estrategias para el control del caracol dirigidas a erradicar la enfermedad.

Métodos: el área pantanosa que rodea el lago Poyang se dividió aleatoriamente en una cuadrícula de 200 m x 200 m utilizando el programa informático ArcGIS, con el que se seleccionaron los recuadros para el estudio de forma aleatoria. El estudio del caracol se llevó a cabo en todos los recuadros seleccionados empleando un marco muestral de 50 m x 50 m y utilizando uno de los bordes de cada cuadro como línea de partida. Se utilizaron no menos de diez marcos en cada recuadro estudiado, cada uno con registros del sistema de posicionamiento global (GPS, por sus siglas en inglés). Se recolectaron todos los caracoles de cada uno de los marcos para determinar el nivel de infección bajo el microscopio. Los datos de altitud de todos los marcos se extrajeron de un mapa topográfico del fondo del lago, para así analizar la altitud media. Se recogieron todos los datos topográficos del caracol y se analizaron estadísticamente con el programa SPSS 20.0 para poder determinar la diferencia del porcentaje de recuadros con caracoles vivos y la densidad media de caracoles vivos en las diferentes zonas del Lago Poyang. Posteriormente, se identificó la altitud de los terrenos pantanosos infestados de caracoles y de las madrigueras de caracoles.

Resultados: se estudió un total de 1.159 recuadros muestrales potenciales de caracoles, de los cuales se investigaron 15.231 marcos (0,1 m^2/marco). 1.241 marcos tenían caracoles *Oncomelania* vivos, lo cual equivale a un 8,15 % del número total de marcos. La densidad media de caracoles vivos era de 0,463/0,1 m^2, con un máximo de 57 caracoles por marco. El porcentaje de marcos con caracoles en el sector meridional (8,13 %) del lago Poyang no fue distinto estadísticamente del porcentaje del sector septentrional (8,21 %). Sin embargo, la densidad media de caracoles vivos en el sector septentrional (0,164/0,1 m^2) del lago fue estadísticamente superior (F= 6,727; P= 0,010) a la del sur (0,141/0,1 m^2). En el sur del lago, la elevación de las zonas pantanosas habitadas por caracoles estaba comprendida entre los 11 y los 16 m, y podía subdividirse aún más en dos cinturones con concentración de caracoles a los 12-13 m y a los 15-16 m de elevación respectivamente. En el norte del lago, la elevación de las zonas pantanosas habitadas por caracoles estaba comprendida entre los 9 y los 16 m. La zona de concentración de caracoles se encuentra a una elevación de 12-14 m.

Conclusiones: la elevación de las zonas pantanosas infestadas de caracoles de la región del lago de Ponyang va de los 9 a los 16 m. La distribución y el hábitat del caracol se han desplazado hacia el norte del lago y hacia una altitud menor, debido a cambios en el nivel del agua tras el cierre de la presa. Sobre la base de las características geológicas actuales del hábitat del caracol, la aplicación focalizada de molusquicidas debería hacerse en las zonas norte con una alta densidad de caracoles, las cuales tienen tráfico bovino y humano frecuente. Centrarse en estos focos de transmisión identificados contribuirá a los esfuerzos de eliminación.

Translated from English version into Spanish by Raquel Acosta, proofread by Mayra León, through

TRANSLATORS
WITHOUT BORDERS
